# Injection Molding Process Simulation of Polycaprolactone Sticks for Further 3D Printing of Medical Implants

**DOI:** 10.3390/ma15207295

**Published:** 2022-10-18

**Authors:** Krzysztof Formas, Anna Kurowska, Jarosław Janusz, Piotr Szczygieł, Izabella Rajzer

**Affiliations:** Department of Mechanical Engineering Fundamentals, Faculty of Mechanical Engineering and Computer Science, University of Bielsko-Biala, 43-309 Bielsko-Biala, Poland

**Keywords:** injection molding, polycaprolactone, process simulation, filament, biomaterials

## Abstract

The aim of the present study was a simulation of the injection molding process of polycaprolactone filament sticks for further 3D printing of osteochondral implants. Polycaprolactone data are not available in the data banks of popular injection molding simulation programs. Therefore, thermal and rheological data from the literature were imported to the material database of Solidworks Plastics software to simulate the injection molding process of filament sticks. The influence of several injection molding parameters including melt temperature, injection time, and injection pressure on the geometry of filament stick (final part) was investigated. Based on the results of the performed simulation and analyses, it was possible to improve the injection process parameters. The accuracy of simulation predictions, based on the literature data, demonstrates the potential of using simulation as a tool to develop polycaprolactone parts for future implants and to optimize the injection molding process.

## 1. Introduction

Facial injuries are a growing clinical problem not only for patients, but also for laryngologist and plastic surgeons. The main cause of injuries is various types of accidents, as well as diseases, congenital deformities, or cancer of supporting tissues such as bone and cartilage. Treatment of osteochondral defects remains a great challenge for many reconstructive surgical procedures.

Three-dimensional (3D) printing is revolutionizing and enhancing the medical industry as it allows for the personalization of the treatment to match a patient’s individual needs. FDM is a 3D printing techniques and offers great flexibility in the handling and processing of materials. In this method, implants are created through the layer-by-layer deposition of a thermoplastic polymer using a 3D printer. The polymeric filament is loaded into the 3D printer, heated above its melting point, softened, and extruded through the nozzle. Usually, the polymers used for biomedical applications are very expensive. Filament materials are generally supplied in spools that are not entirely used during the production of small implants for cartilage or bone replacement [1]. In order to produce filament without such a consumption of biomedical materials, the idea of small sticks that can be joined together was developed by the authors. Moreover, this will provide us an opportunity to put together differently modified sticks and produce personalized implants using a commercially available 3D printer. For their production, injection molding can be applied. The results of the injection molding simulation process presented in this paper will also help us to set the production parameters for the polymer modified with various additives such as hydroxyapatite, bioglass, graphene, drugs, or antibiotics. Therefore, soon, instead of buying entire filament spool, it will be possible to buy a set of differently modified sticks and organize them depending on the patient’s requirements, using a 3D printer. We will be able to produce various scaffolds without having to change the spool as it is in the traditional FDM process. The sticks developed during our project are designed to be used in commercially available printers without any hardware or software modification. 

Injection molding (IM) is a method used to obtain products by injecting molten polymers into the mold cavity with high pressure and then cooling and solidifying them. When the specimen has cooled down, the mold opens and the solid part is ejected, before the mold closes, starting the cycle again [2]. IM is able to manufacture products with precise dimension steadiness, low manufacture cycles, and low costs. Compared with other techniques, injection molding will be found to feature very small tolerances. Therefore, it is also widely used for the production of medical parts. Having fabricated a few sets of filament sticks, it will be possible to join them and obtain filament that can be used in standard or mid-range FDM 3D printer [1]. The flow of molten thermoplastic material in the runners and cavity during the injection molding process is a complex phenomenon that depends on various factors. The viscosity of polymer melt, polymer flow rate, melt temperature, and wall thickness all contribute to the behavior of polymer melt flow [3]. Moreover, the processing parameters also have a great impact on the final properties of the molded product, i.e., filament stick. The changes in processing conditions generate changes in molecular orientation, residual stress, molecular degradation, and the degree of crystallinity [3]. The control of the injection molding machine and the design of the mold are a great challenge for the manufacturer. The lack of knowledge about the material and process conditions leads to molding of parts with some critical defects such as shrinkage and warpage [4]. The type of polymer and its structure affect the shrinkage; however, most of those factors arise from process conditions, such as the injection time, packing time, melt temperature, mold surface temperature, and packing pressure. Warpage can be a result of non-uniform packing pressures in the mold cavities, non-uniform molding temperatures or cooling rates, and non-uniform wall thicknesses [4,5]. When it comes to product quality in injection molding, the control of process parameters is fundamental to create a stable manufacturing process and high quality parts. In the industries, the trial and error method is normally practiced to achieve a desirable product; however, it is a very time-consuming method. Therefore, nowadays, computational simulation of injection molding processes has been widely used. It is a powerful tool that can be used in the early stages of part development to identify and correct mistakes in injection molds and to avoid the risks of costly re-engineering. The commercial CAE packages such as Autodesk Moldflow, Moldex3D, and Solidworks Plastics are used to eliminate the traditional trial and error approach and can provide useful assistance for the optimization of process parameters [6]. However, there are not many types of research for injection molding simulation of the biomedical parts and the material data for biomedical polymers are seldom available from data banks of simulation tools [4]. 

Polycaprolactone (PCL) is a semi-crystalline aliphatic polyester with high biocompatibility, approved by the FDA [7,8]. Polycaprolactone is non-toxic and biodegradable; the good solubility of PCL, as well as its low melting point (59–64 °C), long-term degradation properties (>24 months to lose total mass), and exceptional blend-compatibility, have stimulated extensive research into its potential application in the biomedical field [9]. PCL displays a high viscosity in the melt phase. Therefore, elevated temperatures (above 140 °C) are required to process it [10]. PCL has been used for various biomedical applications such as controlled drug delivery system, tissue-engineering scaffolds, and wound healing [11]. As we mentioned, PCL has a longer degradation time than other polymers from the group of aliphatic polyesters, thus biomaterials made using this polymer can be implanted in areas subjected to increased load [12]. The use of PCL, which has better mechanical parameters, to make scaffolds for the treatment of osteochondral defects allows for maintaining sufficient space for extracellular matrix formation, not only until the moment of producing new tissue, but until it obtains the required mechanical parameters [13]. Unfortunately, PCL data are not available in the data banks of popular injection molding simulation programs. Therefore, in this study, we have create polycaprolactone material data from the literature and use Solidworks Plastic software to simulate the process of injection molding in order to evaluate the influence of operational conditions and mold design on the efficiency of an injection process used to produce PCL filament stick for further 3D printing of facial implants. Thermal and rheological data from the literature were imported to the material database of Solidworks Plastics software for simulating the injection molding process of filament sticks. The influence of several injection molding parameters including melt temperature, injection time, packing time, and packing pressure on the geometry of final part (polymer sticks that will be used for 3D printing) was investigated.

## 2. Materials and Methods

Solidworks Plastics was used for the process of polymer injection simulation and material model building. The material model was created based on PCL polymer CAPA^®^ 6500 with a molecular weight of 50 kDa. All thermal and rheological data necessary for stimulation were collected from the literature and imported to the material database Solidworks Plastics software. CAD Siemens NX was used to build the CAD model of the filament sticks.

In order to verify the simulation process, a Babyplast 6/10P injection molding machine was used to make PCL stick filaments. Chrome-molybdenum 1.2316 X38CrMo16 cold work tool steel was used to make the mold. The size of the mold was 75 mm × 75 mm × 76 mm (height) and the dimensions of the molded part were 60.6 mm (along the sticks) × 57.8 mm (across the sticks) × 23 mm (height).

### 2.1. Material Data Collection

The viscosity of polymers is a very important property in determining the rheological nature of plastics. In order to accurately predict the viscosity of PCL polymer with respect to shear rate, the cross-WLF viscosity model was used. The cross-WLF viscosity model describes the temperature, shear rate, and pressure dependency of the viscosity for thermoplastic materials. The viscosity with the corresponding shear rate is determined through the following formulas:

(1) $$\eta =\frac{\eta_{0}}{1+{\left(\frac{\eta_{0}\dot{\gamma }}{\tau^{*}}\right)}^{1-n}}$$

(2) $$\eta_{0}=D_{1}\exp\left[-\frac{A_{1}\left(T-T_{\mathrm{ref}}\right)}{A_{2}+\left(T-T_{\mathrm{ref}}\right)}\right]$$

where η is the melt viscosity, Pa∙s; η_0_ is the zero viscosity, Pa∙s; $\dot{\gamma }$ is the shear rate, s^−1^; τ^∗^ is the critical shear stress, Pa; n is the power law index, dimensionless; T is the temperature, K; and D1, A1, A2, and T_ref_ are the parameters of the cross-WLF equation.

T_ref_ = D_2_ + D_3_·P (3)

**Figure 1:**
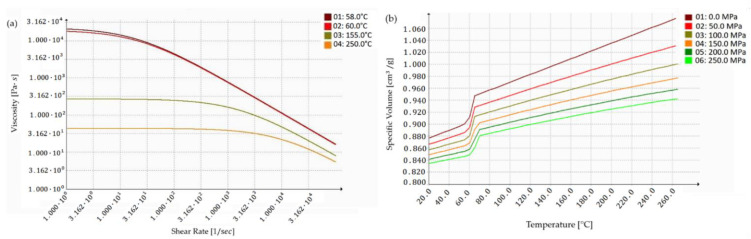
(**a**) Viscosity–shear rate plot (based on cross model data); (**b**) temperature-specific volume plot (based on Tait equation data).

D_2_ and D_3_ are model coefficients determined from experimental measurements for a specific polymer. In most cases, the effect of pressure on viscosity is neglected; therefore, D_3_ is considered to be zero, meaning that the pressure influence is ignored. The cross-WLF viscosity model use the materials’ constants (data-fitted constants) along with predetermined values of temperature and shear rate to calculate the viscosity at a required point. The rheological parameters values were selected on the basis of the literature [14,15] (Table 1) and imported to the SolidWorks program to generate the cross-WLF viscosity model (Figure 1a). The thermal characteristics of the polycaprolactone were obtained from the literature [16,17,18] and are reported in Table 1. The information contains the following: transition temperature, melt flow rate (MFR), thermal conductivity, and melting point. Physical and mechanical properties of polycaprolactone such as solid density, tensile modulus, yield stress, and Poisson’s ratio were also obtained from the literature and are reported in Table 1 [18,19,20].

The simulation of the injection molding process requires knowledge of the time and temperature dependence of the material density at the pressures that occur during the process [21]. The quantity of shrinkage in the injection molded part is a direct function of PVT behavior [22]. Therefore, the pressure–volume–temperature (PVT) relationship is the key rule to characterize the change in density with respect to pressure and temperature [23]. For PVT, we used the Tait model:

(4) $$\upsilon \left(T,\;p\right)=\upsilon_{0}\left(T\right)\left[1-Cln\left(1+\frac{p}{B\left(T\right)}\right)\right]+\upsilon_{t}\left(T,p\right)$$

where C = 0.0894

(5) $$\upsilon_{0}\left(T\right)=\{\begin{array}{c} b_{1m}+b_{2m}\left(T-b_{5}\right)\;\;if\;T>T_{t}\;\\ b_{1s}+b_{2s}\left(T-b_{5}\right)\;\;if\;T<T_{t} \end{array}$$

(6) $$B\left(T\right)=\{\begin{array}{c} b_{3m}exp\left[-b_{4m}\left(T-b_{5}\right)\right]\;\;if\;T>T_{t}\;\\ b_{3s}exp\left[-b_{4s}\left(T-b_{5}\right)\right]\;\;if\;T<T_{t} \end{array}$$

(7) $$\upsilon_{t}\left(T,p\right)=\{\begin{array}{c} \;0\;\;\;\;\;\;\;\;\;\;\;\;\;\;\;\;\;\;\;\;\;if\;T>T_{t}\;\\ b_{7}exp\left[b_{8}\left(T-b_{5}\right)-b_{9}p\right]\;if\;T<T_{t} \end{array}$$

(8) $$T_{t}\left(p\right)=b_{5}+b_{6}\cdot p$$

where *v*_0_ is the specific volume at zero pressure; *b*_1_ (*b*_1*m*_, *b*_1*s*_) and *b*_2_ (*b*_2*m*_, *b*_2*s*_) are the parameters to describe the dependence of *v*_0_ on pressure and temperature; *B* is the sensitivity to pressure and a function of temperature alone with two material constants *b*_3_ (*b*_3*m*_, *b*_3*s*_) and *b*_4_ (*b*_4*m*_, *b*_4*s*_); *v*_t_ is the specific volume decrease due to crystallization; *T*_t_ is the transition temperature; *b*_5_ and *b*_6_ are parameters that describe the change of *T*_t_ with pressure; and *b*_7_, *b*_8_, and *b*_9_ are particular parameters of semi-crystalline polymers that describe the form of the state transition [23,24].

All parameters are presented in Table 2 and were generated using SolidWorks Plastic based on plot (Figure 1b).

### 2.2. Injection Molding Simulation

**Figure 2:**
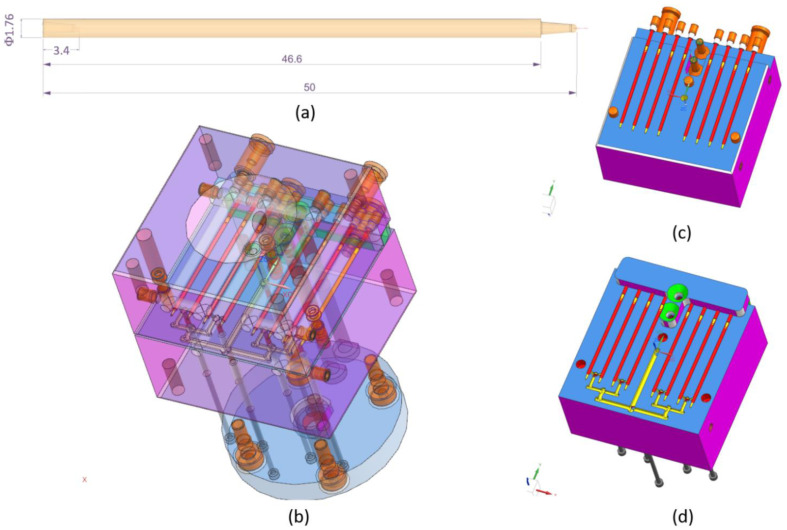
(**a**) The part model; (**b**) injection mold CAD data translucency view; (**c**) injection mold: major CAD data—upper side; (**d**) injection mold: major CAD data—lower side.

**Figure 3:**
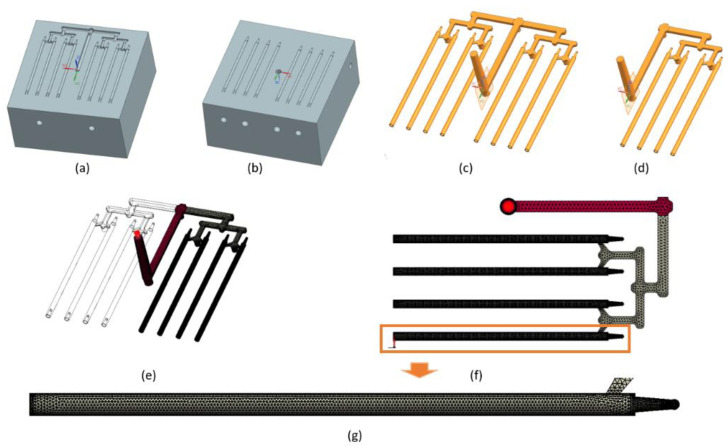
Injection mold, simplified CAD data: (**a**) lower side and (**b**) upper side. Injection part: (**c**) fully CAD data and (**d**) simplified CAD data—one side. 3D mesh simulation model: (**e**) ready to analyze (injection location is visible); (**f**) view from above; and (**g**) stick region.

Simulations were carried out using SolidWorks Plastics 2021 software 29.3.0.0059, Dassault Systemes, Pars, France. The complete project of filament mold based on designed part was executed in the CAD Siemens NX (computer-aided design). An image of the filament model and the CAD model of injection mold are shown in Figure 2. Based on the full CAD assembly of the mold, the simplified CAD model was built. Additional parts such as the inset, plugs, and cooling channels were deleted or transformed to one solid geometry. Figure 3a,b shows the upper and the lower side of the simplified CAD model. It was used to isolate the solid geometry of the injection part. The model of the part was isolated from the simplified mold model as a result of the use of Boolean operations (geometry of solids intersection) (Figure 3c). Figure 3d shows the simplified injection part model with only one side (four sticks). The aim of this simplification was to obtain a shorter calculation time. This operation was possible because the part is symmetrical. All models were prepared in CAD software.

Based on the CAD model of the injection part, the mesh model was developed. The mesh subdivides domains of the simulation into discrete cells (in this case, the runner system and cavity area). The solid mesh procedure subdivides the volume of each domain into polyhedral-shaped elements. These solid elements have a tetrahedral shape. The software creates a surface mesh too, but the surface elements (faces) represent the boundaries of the closed solid polyhedral elements. A solid mesh is suitable for models with a complex geometry. The mesh model was prepared using injection molding simulation software. Figure 3e shows the mesh model of the injection part. Table 3 contains the mesh parameters. Figure 3f shows the runner system and sticks area of the injection part. The mesh of sticks is more precise (higher number of tetrahedral elements). This means the analysis results will be more accurate. Figure 3g presents the discretization of stick volume.

**Figure 4:**
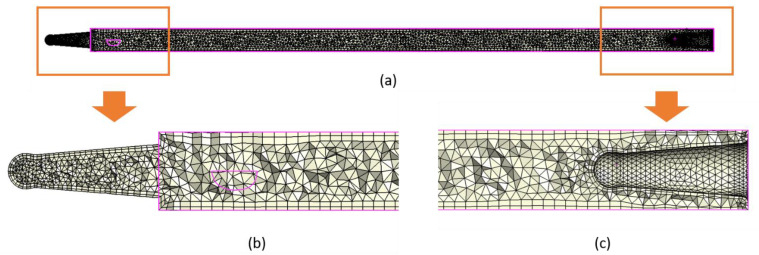
Three-dimensional (3D) mesh model of stick: (**a**) clipping section; (**b**) external joint region (boundary layers are visible); and (**c**) internal joint region (boundary layers are visible).

Figure 4 shows the longitudinal section through the mesh model of stick. Figure 4b,c presents the joint regions of sticks. The areas are very small (0.8–1.2 mm diameter). Discretization in these areas is much more precise. Additionally, two external boundary layers were manually added at the outside of sticks for better accuracy of calculations.

**Figure 5:**
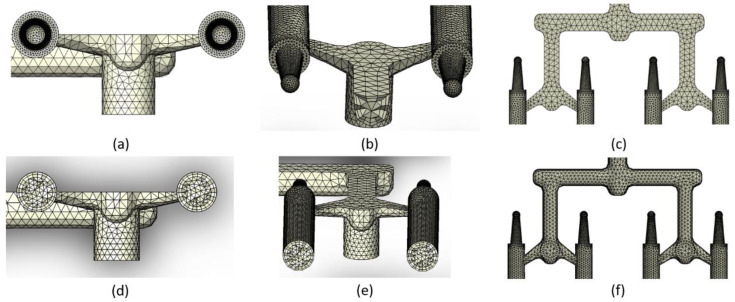
Three-dimensional (3D) mesh model of a stick: (**a**) front view of sticks; (**b**) back view of stick; and (**d**,**e**) clipping section (boundary layers are visible). Sticks and distribution runners: (**c**) view from above and (**f**) bottom view.

Additionally, two external boundary layers were manually added at the outside of the sticks. The nominal thickness of a single layer was set in the software at 0.04 mm. As we see in the mesh model, the layers thickness is not regular. The true thickness of the layers is determined by the geometry of the CAD model (especially narrow areas). Their shape and size are also influenced by the other discretization elements. By adding external boundary layers, it will be possible to obtain more accurate results (better accuracy of calculations); for example, better quality of the temperature distribution result on the workpiece outer surface. Figure 5a,b presents the front and back views of sticks. Figure 5d,e presents the cross section through the mesh model of a stick. Two external boundary layers are visible. Figure 5c,f shows the distribution runners. 

In the next step, the process parameters based on our previous works were established [1,25]. The process parameters (mold temperature, melt temperature, and injection pressure) are presented in Table 4. 

## 3. Results

**Figure 6:**
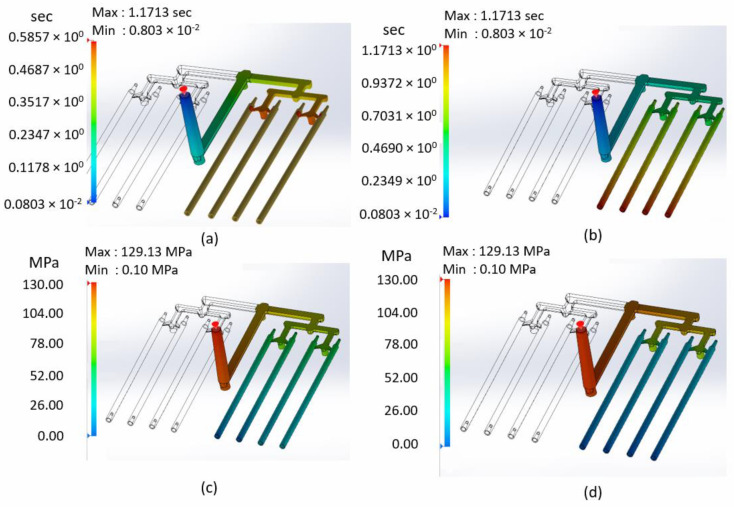
(**a**) Fifty percent of the fill time; (**b**) total fill time; (**c**) pressure at the end of fill; and (**d**) pressure at the end of packing.

Figure 6a shows fifty percent of filling time (about 0.6 s). Until this time, all runner systems were filled. Liquid material was starting to flow to the sticks. Figure 6b presents the total fill time (about 1.2 s). After that time, the entire volume of the part was filled. The results of the fill time analysis indicate that the entire capacity of the sticks was filled evenly. 

Figure 6c shows the pressure at the end of fill. Pressure falls evenly from the maximum values closer to the injection point to zero at the end sticks. Figure 6d shows the pressure at the end of packing. The greatest pressure drop is visible after material flows from distributing runners to sticks (through small gates). It is important to remember the PIR (pressure intensification ratio). The value of injection pressure from the analysis could be higher than the real machine pressure during the real injection process. The PIR value is the relationship between the injection pressure and the hydraulic pressure (oil pressure in the piston of the injection molding machine). In practice, the pressure at the machine could be much lower than the injection pressure. A typical range of PRI value is between 7 and 15. Therefore, during the injection process, pressure should be set experimentally depending on the visual quality assessment of the part.

**Figure 7:**
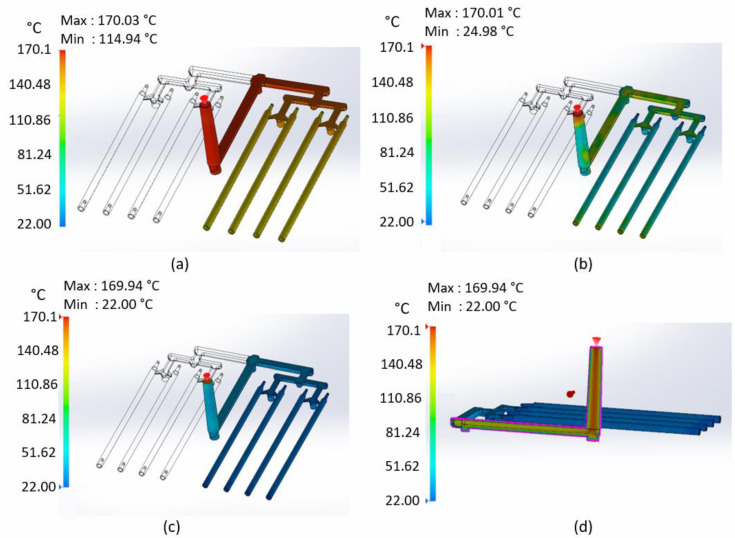
(**a**) Flow front temperature; (**b**) temperature at the end of fill—boundary surface; (**c**) temperature at the end of packing—boundary surface; and (**d**) temperature at the end of packing—clipping section through the runner.

Figure 7a shows the temperature at the flow front. The highest value of temperature (170 °C) is visible near the injection point. This value is assumed as the flow temperature in the injection process. Temperature drops further from the injection point. This is because of the heat exchange between liquid plastic and mold at an ambient temperature (22 °C). The even temperature distribution in the process is noticeable. Each stick is filled at the same temperature. It is important for the high quality of the finished part and to maintain consistent properties of each stick.

Figure 7b shows the temperature at the end of the fill. The temperature distribution is irregular. Some regions of the part have reached the solidification temperature. The remaining places are still hot (plastic is in a liquid state). After this stage, material flow is still possible. Figure 7c shows the temperature at the end of the packing. All boundary areas of sticks reached an ambient temperature (22 °C). Most of runner’s areas did so too, except for a few regions (wider gates of main and distribution runners), where the temperature is higher. However, the entire outside boundary surface is in a solid state. Figure 7d shows the temperature at the end of the packing inside of the main runner. Only the outer walls are in contact with the cold surfaces of the mold. Inside the part, heat transfer is slow, so the core is still very hot (above 120 °C). 

**Figure 8:**
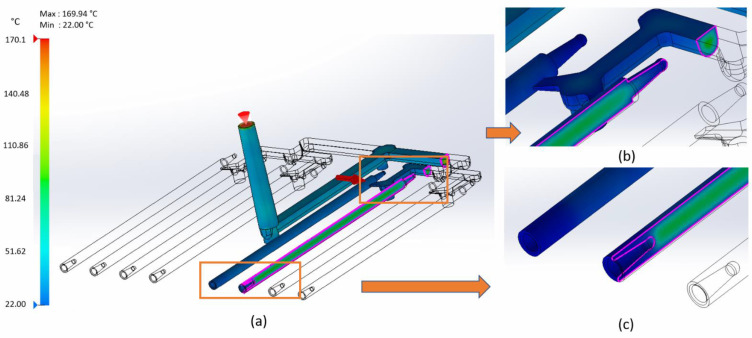
(**a**) Temperature at the end of packing—clipping section through the stick; (**b**) external joint areas; and (**c**) internal joint areas.

Figure 8 shows the temperature at the end of the packing inside of the part. The temperature inside the sticks is still high (about 70–90 °C). After this stage, there is an ejection from the mold. The interior of the part cools in the open air.

**Figure 9:**
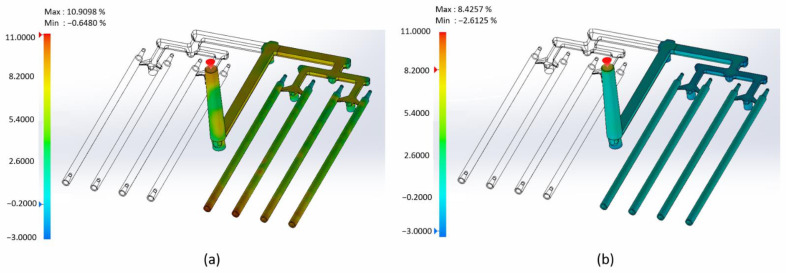
(**a**) Volumetric shrinkage at the end of fill; (**b**) volumetric shrinkage at the end of packing.

Figure 9a shows the volumetric shrinkage of the part at the end of the fill. The results of the analysis indicate that the shrinkage at the end of fill in most areas is large. Especially at the end of sticks, it is about 10 percent. During the filling process, the material cools down. Under these conditions (temperature drop, reaching the solid form), volumetric shrinkage takes place. It must be compensated by additional material from the piston of the injection molding machine (pack stage). Figure 9b presents volumetric shrinkage at the end of packing. At the sticks, shrinkage does not exceed 2 percent.

**Figure 10:**
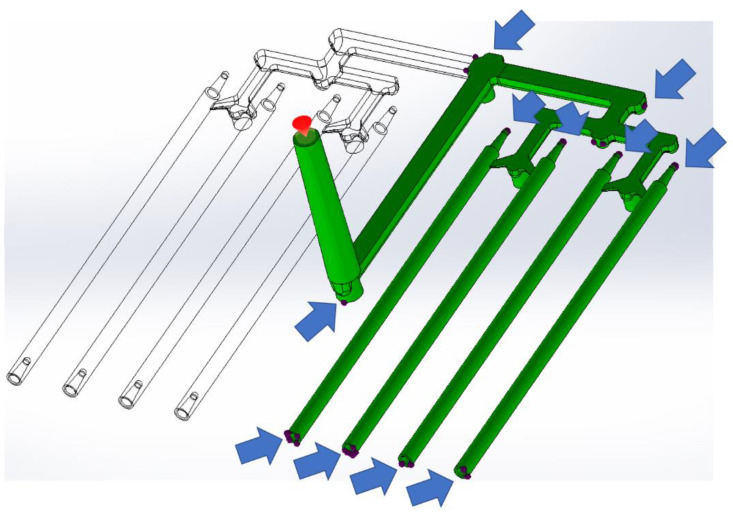
Air traps.

Figure 10 shows places where defects, such as air bubbles and empty cavities, may occur. The most risky regions are located at both ends of the sticks (internal and external joint areas).

**Figure 11:**
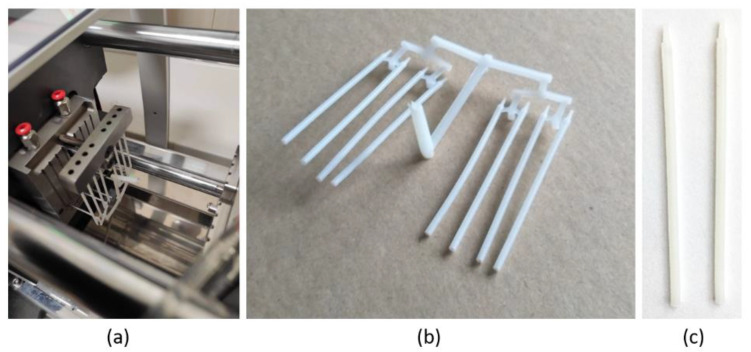
PCL stick produced using parameters developed during the simulation: (**a**) stick at the end of the injection molding process; (**b**) produced part; and (**c**) PCL filament sticks.

In order to verify the simulation process, a Babyplast 6/10P injection molding machine was used to make PCL filaments in the form of sticks using the mold and parameters used previously for simulation. The shape of the mold was properly reproduced and good quality sticks were obtained (Figure 11).

## 4. Discussion

Achieving high-quality parts is a crucial task for the injection molding of sticks for further 3D printing of implants. With computer simulation, potential defects, such as air trap, weld line positions, and sink mark, emerging during the injection molding process can be predicted, thus mistakes in the design can be modified without excessive expenses of time and money [4]. With thermoplastics injection molding simulation, the material data are generally available from the simulation tools. Users could easily choose thermoplastics material data to simulate entire phases of the thermoplastics injection molding process [26]. However, polycaprolactone data are not available in the data banks of common injection molding simulation programs. Therefore, in this study, we created polycaprolactone data from the literature. The basic material properties are listed in Table 1. The selected materials properties were implemented in Solidworks Plastic software to simulate the process of injection molding. We designed the mold and evaluated the influence of the injection time, pressure, and temperature on the geometry of the part. Our studies demonstrated that the selected parameters had a good effect on the quality of the part (sticks). The mold filling process was correct. The material temperature was almost the same in the entire sticks area. The entire capacity of sticks was filled evenly and shrinkage did not exceed 2 percent. In our previous work, we started to develop the idea of filament stick production by injection molding; however, the parameters of the process were selected using the traditional trial and error method [1]. Then, computer-aided engineering was used to verify the part and mold construction [25]. The simulation of the injection process showed the faults and imperfections of the former injection mold and sticks’ design. The sticks were arranged in series; therefore, the flow path of the material to those more distant from the injection site was much longer and the last stick was filled with a lower pressure than the others in the series. The problem of temperature and pressure drop was caused by the narrow gates near the sticks. The negative impact of their small cross section was also visible in the simulation of the injection time. After passing through them, the material propagated much slower. The changes introduced in the newly designed sticks and the design of a new mold as a result of the simulation presented in this work allowed us to obtain a new quality of sticks.

## 5. Conclusions

Our studies indicate that SolidWorks software can be used as a simulation tool for investigating the stick quality. The parameters selected during the simulation were applied in a real process using the injection molding machine. The good results of the simulation were confirmed by the good quality of the obtained stick. Comparisons of the results of simulation and experimental methods show that SolidWorks offers similar results. Our study has shown the most significant parameters of the injection molding process, such as the injection time, pressure, and temperature. By combining the experimental results and simulation methods, it is possible to obtain a qualitative prediction of the morphological characteristics of the polymer obtained with various additives. Therefore, the promising results of these preliminary studies encourage us to continue working on the modification of PCL. Different additives (bioactive powders, antibacterial particles, and drugs) will be used in the future to study the effect of the mentioned process parameters on the modified PCL polymer. Moreover, other simulation software will be used to perform a verification of the obtained results. 

## 6. Patents

Application number: P.428429; Filing date: 31 December 2018. Patent number: Pat.240243. Date of granting the right: 12 September 2021. Authors: Izabella Rajzer, Adam Jabłoński, Anna Kurowska, Jerzy Kopeć, and Marcin Sidzina. “A method of producing implants from bioresorbable thermoplastic polymer composites, especially in the form of three-dimensional scaffolds, intended for the reconstruction of cartilage and bone tissue defects, using 3D printing”. 

## Figures and Tables

**Table 1:** Rheological, thermal, and mechanical properties of polycaprolactone needed to build the model, collected from the literature.

Parameter	Value	Literature
Rheological properties. Viscosity model: Cross-WLF		
D_2_ [K]	243.15	[10,14,15]
n	0.1547	
D_1_ [Pa∙s]	2.21915 × 10^12^	
τ* [Pa]	429,551	
A_1_	29.20632	
A_2_ [K]	51.6	
Physical Properties		
Solid density, kg/m^3^	1140	[19]
Thermal Properties		
Glass transition, °C	−60	[16,17,18]
Specific heat, [J/kgK]	2100	
Melt flow rate (MFR), g/10 min (190 °C/2.16 kg)	28	
Thermal conductivity, [W/m·K] (20 °C)	0.1	
Melting point [°C]	60–62	
Mechanical properties		
Yield stress, σ_y_, [MPa]	17.5	[20]
Tensile modulus [MPa]	430	[18]
Poisson’s ratio	0.44	[20]

**Table 2:** Pressure–volume–temperature properties [21].

P–V–T (Pressure–Volume–Temperature) Properties 13-Parameter Modified Tait Equation	
**Parameter**	**Value**
b_1m_ [m^3^/kg]	9.463 × 10^−4^
b_2m_ [m^3^/(kg-K)]	6.514 × 10^−7^
b_3m_ [Pa]	2.030180 × 10^8^
b_4m_ [1/K]	4.521 × 10^−3^
b_1s_ [m^3^/kg]	9.045 × 10^−4^
b_2s_ [m^3^/(kg-K)]	6.291 × 10^−7^
b_3s_ [Pa]	2.52593 × 10^8^
b_4s_ [1/K]	7.368 × 10^−3^
b_5_ [K]	336.77
b_6_ [K/Pa]	1.497 × 10^−8^
b_7_ [m^3^/kg]	4.181 × 10^−5^
b_8_ [1/K]	4.042 × 10^−1^
b_9_ [1/Pa]	7.393 × 10^−9^

**Table 3:** Discretization details.

Mesh Details	
solver type	AMG (algebraic multigrid)
mesh type	solid mesh
mesh volume, [mm^3^]	955
mesh type	tetrahedral hybrid
total elements of surface mesh	59,756
total nodes of surface mesh	29,857
total elements of solid mesh	394,602
total nodes of solid mesh	119,344
analyze type	fill and pack

**Table 4:** Manufacturing parameters.

Process Details	
Process Parameter	Value
ambient temperature, [°C]	22
melt temperature, [°C]	170
mold temperature, [°C]	22
filling time, [sec]	1
injection pressure at the nozzle (filling), [MPa]	130
packing time, [sec]	2.5
injection pressure at the nozzle (packing), [MPa]	20
cooling time, [sec]	25

## Data Availability

Data are contained within the article.
